# Combining QSAR Modeling and Text-Mining Techniques to Link Chemical Structures and Carcinogenic Modes of Action

**DOI:** 10.3389/fphar.2016.00284

**Published:** 2016-08-30

**Authors:** George Papamokos, Ilona Silins

**Affiliations:** ^1^Department of Physics and School of Engineering and Applied Sciences, Harvard UniversityCambridge, MA, USA; ^2^Department of Physics, University of IoanninaIoannina, Greece; ^3^Biomedical Research Division, Institute of Molecular Biology and Biotechnology Foundation for Research and TechnologyHeraklion, Greece; ^4^Institute of Environmental Medicine, Karolinska InstitutetStockholm, Sweden

**Keywords:** carcinogens, mode of action, text mining, QSAR, risk assessment, toxicity, prediction

## Abstract

There is an increasing need for new reliable non-animal based methods to predict and test toxicity of chemicals. Quantitative structure-activity relationship (QSAR), a computer-based method linking chemical structures with biological activities, is used in predictive toxicology. In this study, we tested the approach to combine QSAR data with literature profiles of carcinogenic modes of action automatically generated by a text-mining tool. The aim was to generate data patterns to identify associations between chemical structures and biological mechanisms related to carcinogenesis. Using these two methods, individually and combined, we evaluated 96 rat carcinogens of the hematopoietic system, liver, lung, and skin. We found that skin and lung rat carcinogens were mainly mutagenic, while the group of carcinogens affecting the hematopoietic system and the liver also included a large proportion of non-mutagens. The automatic literature analysis showed that mutagenicity was a frequently reported endpoint in the literature of these carcinogens, however, less common endpoints such as immunosuppression and hormonal receptor-mediated effects were also found in connection with some of the carcinogens, results of potential importance for certain target organs. The combined approach, using QSAR and text-mining techniques, could be useful for identifying more detailed information on biological mechanisms and the relation with chemical structures. The method can be particularly useful in increasing the understanding of structure and activity relationships for non-mutagens.

## Introduction

Cancer is a major public health problem and the number of cases are expected to increase in the future (Frankish, 2003). Current research indicates that environmental factors, including chemicals, have a major role in the disease development, emphasizing the importance to prevent exposure to compounds possessing carcinogenic potential (Christiani, 2011; Landrigan et al., 2011; Wu et al., 2016). Traditionally, the animal bioassay has been the main method used to identify carcinogens. However, these tests are costly and time-consuming, and recent regulatory policies require a reduction in the number of animals used in chemical testing. Consequently, there is a need for alternative methods to examine toxicological effects of chemicals (Pelkonen, 2010).

In order to develop reliable non-animal based tests to identify carcinogens, knowledge of the biological mechanisms that lead to cancer is required. For example, the understanding of chemicals’ modes of action (MOA), i.e., the sequence of key events resulting in cancer, has become increasingly important in hazard identification and risk assessment (Sonich-Mullin et al., 2001; US-EPA, 2005; Boobis et al., 2008). The current understanding of how chemicals cause cancer involves two major MOAs: genotoxicity and non-genotoxicity (indirect genotoxicity). A genotoxic MOA means that the chemical interacts directly with the DNA (which can result in mutagenicity), whereas a non-genotoxic MOA denotes indirect effects, such as stimulation of cell proliferation or inhibition of cell death (US-EPA, 2005).

The huge collection of biomedical articles in MEDLINE, available through the search engine PubMed^1^, provides a great source of information for researchers to utilize and generate new knowledge. However, considering the enormous amount of articles, more than 26 million to date, it is getting more and more problematic for researchers to handle information relevant to them. For such purposes, techniques like text-mining could be used for locating and managing information overload. Recently, biomedical text-mining has become increasingly popular for handling the large volumes of texts in biomedical sciences (Cohen and Hersh, 2005; Zweigenbaum et al., 2007). Today, there is a wide range of different text-mining tools available to support researchers in the biomedical field (Cohen and Hersh, 2005; Zweigenbaum et al., 2007; Zhu et al., 2013; Fleuren and Alkema, 2015; Gonzalez et al., 2016). One such tool, CRAB, has been developed to support classification of literature relevant to cancer risk assessment (Korhonen et al., 2009, 2012; Guo et al., 2014). This tool automatically classifies literature based on the carcinogenic evidence that is mentioned in the text of scientific abstracts. Based on the text analysis the tool generates toxicological literature profiles that can be used for cancer risk assessment or cancer research. This approach facilitates the detection of new patterns in data, which could be a nearly impossible task by manual literature search and evaluation. Such data patterns can be used, e.g., to compare individual substances or groups of chemicals to generate new hypotheses that can be tested experimentally (Korhonen et al., 2009, 2012; Kadekar et al., 2012; Silins et al., 2014; Ali et al., 2016).

Quantitative structure-activity relationship (QSAR) modeling is an important computational tool in medicinal chemistry and predictive toxicology (Hansch et al., 1962; Cherkasov et al., 2014). It is a procedure by which a chemical structure is quantitatively linked with a clearly defined process, typically biological activity or chemical reactivity. The QSAR model systems build on structure-activity relationships of known chemicals, and can be used to predict the toxicity of unknown chemicals based on their structures (Combes, 2012). This technique has proven especially useful in predicting mutagenicity based on structural alerts, which are mechanistically linked to carcinogenicity (Benigni and Bossa, 2011). Structural alerts are the molecular structures and reactive groups that are responsible for a toxic effect (Benigni et al., 2013). The QSAR method can thus both predict carcinogenicity, and mutagenicity, and provide information about structural alerts based on the chemical structures (Benigni and Bossa, 2006). Traditionally, the QSAR method has been better in predicting reactive (genotoxic) compounds compared to non-reactive (non-genotoxic) carcinogens, however, recently a new set of structural alerts relating to non-genotoxic mechanisms including, e.g., oxidative stress, hormonal imbalance, and peroxisome proliferation has been identified (Benigni et al., 2013).

Several new alternative approaches for predicting carcinogens in connection with QSAR have been suggested (Benigni, 2014). For example, a strategy using QSAR in a tiered approach combined with *in vitro* tests for genotoxicity and tumor promotion has been proposed (Benigni, 2014). Another approach to improve prediction in combination with QSAR is based on mechanistic information, involving the concept of adverse outcome pathways (AOP; Benigni, 2014). The AOP outlines the sequence of events starting from a molecular initiating event, through a series of key events, resulting in an adverse effect (Vinken, 2013). The AOP and the MOA (described above) are similar concepts that take into account mechanistic information to improve, e.g., risk assessment, however, one major difference is that a MOA focuses on the details specific to a particular chemical, whereas the AOPs are chemical-agnostic (Edwards et al., 2016; Kleinstreuer et al., 2016).

The purpose of this study was to test whether combining QSAR methodology with a text-mining approach based on carcinogenic MOA could be useful to identify new associations between chemical structures and biological activities related to carcinogenesis. Ninety-six rat carcinogens were selected from the National Toxicology Program’s (NTP) database, and literature profiles and QSAR data were generated for each carcinogen. Based on both the QSAR data and on text mining-generated literature profiles we found that skin and lung rat carcinogens were mainly mutagenic, while the group of carcinogens affecting the hematopoietic system and the liver also included a large proportion of non-mutagens. Mutagenicity was a found to be a frequently reported endpoint in the literature, however, less common endpoints such as immunosuppression and hormonal receptor-mediated effects were also found in literature on some carcinogens, which could be of potential importance. The approach to combine QSAR and text-mining could be particularly useful for identifying biological mechanisms of potential relevance to non-mutagens.

## Materials and Methods

### Selection of Carcinogens

The NTPs database^2^ was used to select the rat carcinogens included in this study. Four common organ sites were selected, including the hematopoietic system (i.e., leukemia or lymphoma), liver, lung, and skin. All rat carcinogens affecting these four organs and classified by NTP as positive, clear, or some evidence were selected for further analysis. Based on these criteria, a total of 126 rat carcinogens were included. Among these carcinogens, 30 chemicals affected one or more of the other three organs, leaving a total of 96 individual chemicals for further analysis.

### Analysis of Carcinogenic MOA Using a Text-Mining Approach

To investigate the carcinogenic MOAs concerning the 96 selected rat carcinogens we used the text mining-based tool CRAB (Korhonen et al., 2009, 2012; Guo et al., 2014) to analyze the scientific literature. The published literature concerning these carcinogens was retrieved from PubMed^3^ using the chemicals’ nomenclature or CAS numbers. This analysis was based on literature published until January 2015. The literature collection of each carcinogen was automatically classified by the tool, which categorizes scientific abstracts according to a taxonomy that covers the main types of evidence for carcinogenic MOAs. In brief, the taxonomy structure includes two main MOA classes: genotoxicity and non-genotoxicity. It is further branched into 25 sub-categories, ranging from common carcinogenic endpoints, such as mutations, to less common effects, such as inflammation. The classification is based on the evidence mentioned in the abstracts’ text. For each carcinogen of interest the tool generates a publication profile based on the scientific literature, thus the profile reflects the current knowledge about this chemical. The tool automatically calculates the proportion of abstracts in each category (per total number of MOA-relevant abstracts; Guo et al., 2014). The tool is based on advanced text-mining techniques and has shown to generate classification of high accuracy. It can be found at: http://omotesando-e.cl.cam.ac.uk/CRAB/request.html.

The carcinogens were grouped according to their target organ, predicted mutagenicity/non-mutagenicity and structural alert. Literature profiles for each group were generated by calculating the average percent for each MOA subcategory. Carcinogens with less than 10 abstracts were excluded in the text-mining analysis. The statistical significance of the results was calculated using the *t*-test.

### QSAR Analysis

VEGA^4^ Non-Iterative Client (VEGANIC) v1.0.8, a standalone JAVA-based software was employed and three different SAR models were applied to the current dataset: Mutagenicity model CAESAR (Ferrari and Gini, 2010) version 2.1.12, Mutagenicity SarPy model version 1.0.6-DEV (Ferrari et al., 2013), and Benigni–Bossa Mutagenicity (TOXTREE; Benigni et al., 2008) version 1.0.0-DEV. The input structural data of the chemicals were given in SMILES format (Weininger, 1988). The SMILES chemical structures for each compound were retrieved from PubChem, ChemSpider, or Wikipedia databases using CAS registry numbers, IUPAC nomenclature or empirical chemical names. In a first step, the dataset of 96 carcinogens was curated and counter ions, salts and disconnected structures were removed as no identical compounds were located. In total, 75 carcinogens were included in the QSAR analysis.

### Linking QSAR Data with Literature Profiles of Carcinogenic MOA

The results generated from three different SAR models were compiled in order to decide the structures of carcinogens according to Benigni Bossa code (Benigni et al., 2008). Each of the 75 carcinogens analyzed was associated with a structural alert, if given from the QSAR output. Some of the chemicals were mutagens without a structural alert (named here unspecific mutagens) or were predicted non-mutagens (typically without a proposed structural alert).

Certain classification rules were applied when the carcinogens were grouped as mutagenic or non-mutagenic based on the output from the QSAR analysis. When identical results were generated from all the three QSAR models the classification as mutagenic or non-mutagenic was considered certain. If one model presented conflicting results, the experimental result was assumed more reliable than the predicted outcome. As default, carcinogens were considered mutagenic if the QSAR models presented conflicting results (e.g., if one model predicted the chemical as mutagenic and another model as non-mutagenic).

#### Grouping of Chemicals

First, carcinogens were grouped according to their target organs (hematopoietic system, liver, lung, and skin). Secondly, carcinogens were grouped based on the QSAR output for each chemical, as mutagens or as non-mutagens. In cases where a chemical could have been entered into both classes because of conflicting results from the different QSAR models, a decision was made regarding the dominant category, and it was entered into that single class. The two groups (mutagens and non-mutagens) were further associated with their average MOA literature profile, an analysis which included 46 mutagens and 22 non-mutagens. Thirdly, carcinogens were grouped based on their structural alerts; eight groups were formed including mutagens (quinones, primary aromatic amines, nitro aromatics, unspecific mutagens, hydrazine, epoxides, and aziridines and aliphatic halogens) and non-mutagens. For each of these groups an average MOA literature profile was generated.

## Results

### Literature Analysis of Carcinogenic MOA Using the CRAB-Tool

The rat carcinogens affecting the four selected target organs (hematopoietic system, liver, lung, and skin) included in total 126 chemicals. Of these, 30 were carcinogenic in at least one of the other organs, leaving 96 individual rat carcinogens for further analysis. The liver was the most common target site, since 58 of the chemicals affected the liver in rats. Twenty-four chemicals caused cancer in the hematopoietic system, and 22 were skin and lung carcinogens, respectively (**Table 1**). The total literature collection of the selected carcinogens included almost 130 000 scientific abstracts retrieved from PubMed. The group of skin carcinogens was the most well-studied with a literature collection of almost 50 000 abstracts.

**Table 1:** Literature data for carcinogens affecting the hematopoietic system, liver, lung, and skin in National Toxicology Program’s (NTP) 2-year rat bioassays.

Target organ	Number of carcinogens	Number of abstracts (retrieved from PubMed)	Number of abstracts relevant to carcinogenic MOA (modes of action) (CRAB-tool analysis)
Hematopoietic system	24	21,837	4,296
Liver	58	49,862	18,097
Lung	22	6,895	1,648
Skin	22	49,902	6,251

Total	126	128,496	30,292

*The number of carcinogens per target organ, number of abstracts retrieved from PubMed and the number abstracts classified as relevant to carcinogenic MOA for each target organ are shown*.

From the whole abstract collection >30 000 abstracts (∼25% of the whole retrieved literature collection) were classified as relevant for carcinogenic MOA by the CRAB-tool. Liver carcinogens were the most well-studied of the four target organs regarding literature relevant to carcinogenesis and MOAs as shown in **Table 1**.

**FIGURE 1:**
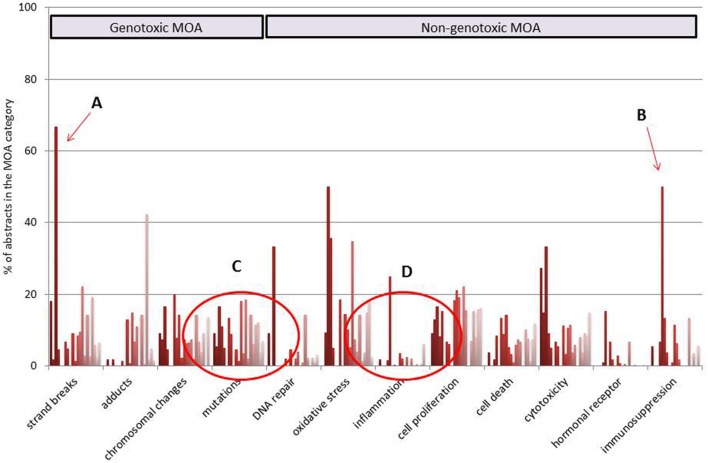
**Individual literature profiles of 21 rat carcinogens of the hematopoietic system**. Twelve selected categories of carcinogenic MOA (modes of action) are shown.

By using the CRAB-tool, the literature collection retrieved from PubMed for each carcinogen was classified, and carcinogenic MOA profiles were generated. As an illustration of a literature distribution pattern, MOA profiles of 21 individual rat carcinogens of the hematopoietic system are shown in **Figure 1**. The figure shows the percent of abstracts relevant to a certain MOA category, for each carcinogen. From the literature distribution it is observed that one of the carcinogens has a large proportion of literature classified in the *strand breaks* category (A) and another carcinogen in the *immunosuppression* category (B). From the same figure can also be seen that the literature of most carcinogens reports about *mutagenicity* (C), but only one carcinogen has a large proportion of the literature classified in the *inflammation* category (D).

**FIGURE 2:**
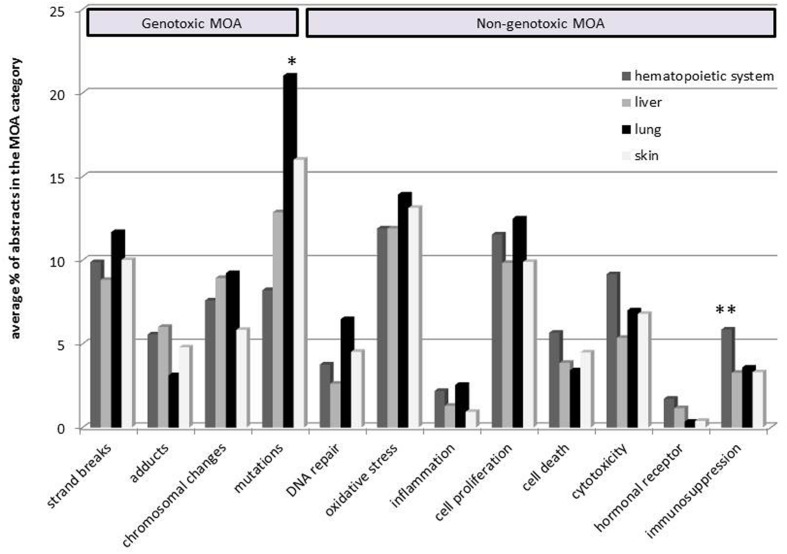
**Comparison of literature profiles for four target organs including hematopoietic system, liver, lung, and skin**. The average percent of abstracts classified in the MOA taxonomy is shown on the y-axis. Carcinogens were grouped according to their target organ(s). ^∗^Significantly different from carcinogens of the hematopoietic system (*p* ≤ 0.05), ^∗∗^Significantly different compared to liver carcinogens (*p* ≤ 0.05).

Chemicals were grouped according to their target organ and literature profiles were generated for each group (**Figure 2**). This approach facilitates comparison of carcinogens affecting different target organs. If a specific MOA category stands out in the comparison it may reflect a potentially important mechanism for this organ. The data patterns showed that a larger proportion of literature concerning lung carcinogens reported about mutations as compared to the other organs (significantly different compared to carcinogens of the hematopoietic system). The figure further shows that carcinogens of the hematopoietic system have a significantly larger proportion of literature classified in the immunosuppression category compared to liver carcinogens. In general, the literature patterns indicated that endpoints such as mutations and oxidative stress were commonly studied, while inflammation and hormonal receptor-mediated effects were less frequently reported in literature.

The literature patterns were analyzed in more details. A compilation of the results from the CRAB literature analysis for the four target organs is shown in **Table 2**. The literature analysis showed that mutation was a commonly studied endpoint, reported in the literature of 80–90% of all carcinogens included. Other common endpoints were chromosomal changes and strand breaks. In addition, mutagenicity was found to be the most well-studied MOA category regarding rat carcinogens of the liver, lung, and skin. Regarding carcinogens of the hematopoietic system, oxidative stress was the most well-studied MOA category, for which, on average, 12% of the MOA literature was classified as relevant.

**Table 2:** Results from the classification of abstracts relevant to carcinogenesis.

Target organ	Most common MOA (percent of all chemicals)	Most well-studied MOA (average percent)
Hematopoietic system^a^	Mutations (90%)	Oxidative stress (12%)
Liver^b^	Mutations (82%)	Mutations (13%)
Lung^c^	Chromosomal changes, mutations, strand breaks (89%)	Mutations (21%)
Skin^d^	Mutations (89%)	Mutations (16%)

*^a^21/24, ^b^49/58, ^c^19/22, ^d^18/22 carcinogens were included in the analysis using the CRAB-tool*.

### Analysis of Carcinogens Using the QSAR Method

The QSAR method was used to predict the type of carcinogen (mutagen or non-mutagen) and structural alerts. When carcinogens were grouped according to their target organ the QSAR data indicated that most skin carcinogens were mutagens (**Table 3**). Grouping of skin carcinogens suggested two dominating structural alerts: aliphatic halogens, epoxides, and aziridines, which are both alkylating and direct-acting chemicals. Most of the lung and liver carcinogens were also predicted mutagens, however, a large proportion (38%) of the liver carcinogens were predicted non-mutagens. In addition, although the majority of carcinogens affecting the hematopoietic system were predicted mutagens, a large part (37%) were classified as non-mutagens (**Table 3**). Thus, compared to carcinogens of the skin and lung, a large proportion of the liver carcinogens and carcinogens affecting the hematopoietic system were non-mutagens.

**Table 3:** The number of carcinogens with predicted structural alerts shown for each target organ.

Structural alert	Hematopoietic cancer	Liver	Lung	Skin
Mutagens: aliphatic halogen	3	3	2	3
Mutagens: epoxides and aziridines	2	0	0	3
Mutagens: hydrazine	1	1	0	0
Mutagens: unspecific	3	3	6	1
Mutagens: nitro aromatics	2	1	3	2
Mutagens: primary aromatic amines	0	5	0	0
Mutagens: quinones	0	4	0	0
Mutagens: other structural alerts	1	8	3	2

Mutagens (in total)	12	25	14	11

Non-mutagens	7	15	4	2

*Some carcinogens affected more than one target organ*.

### Combining QSAR and Text Mining-Generated MOA Profiles

**FIGURE 3:**
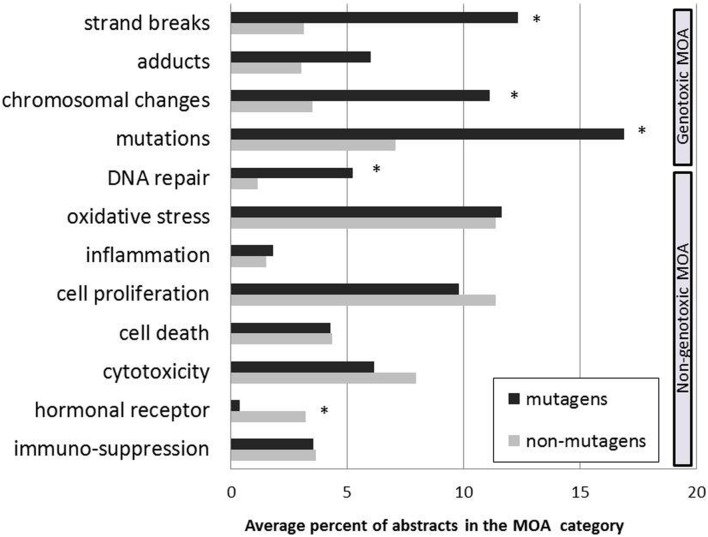
**Distribution of literature concerning mutagens and non-mutagens in the MOA taxonomy**. Carcinogens were grouped into two groups (mutagens and non-mutagens) based on the results from QSAR modeling. Carcinogenic MOA profiles were generated for the two groups. The literature distribution is shown as the average percent of abstracts in the MOA category. ^∗^Signficantly different compared to the other group (*p* ≤ 0.05).

Chemicals were grouped either as mutagens or as non-mutagens, based on the output from the QSAR modeling. The group of mutagens included 46 chemicals and 22 chemicals were non-mutagens. Literature profiles were generated for each of the two groups. **Figure 3** shows the differences in the literature distributions between them. The proportion of literature classified as relevant to genotoxic endpoints or to non-genotoxic categories is in line with the data from the QSAR analysis. For example, literature concerning mutagens was more frequently classified in genotoxic MOA-categories, including mutation, strand breaks, and chromosomal changes. Non-mutagens, on the other hand, had more literature classified in non-genotoxic MOA-categories, e.g., hormonal receptor-mediated effects, as compared to mutagens.

Sixty-eight carcinogens, for which QSAR data had been generated and that had enough literature data required for analysis were grouped based on their structural alerts. The aim was to investigate whether more detailed information regarding the chemical structures could be associated with a particular MOA category. Eight groups were formed, seven groups included mutagens with different structural alerts and one group consisted of non-mutagens (without structural alerts). Each structural alert group was linked to its corresponding literature profile. The two most common MOA categories for each group is presented in **Table 4**. The mutation and oxidative stress categories were the dominating categories. Cell proliferation and oxidative stress were the most common categories for non-mutagens (same data as shown in **Figure 3**). However, the number of carcinogens included in each group was small, ranging from three carcinogens in the group of hydrazines and epoxides and aziridines, to 22 carcinogens in the group of non-mutagens.

**Table 4:** Linking structural alerts with carcinogenic MOA information.

Structural alert	Most common MOA categories
1. Aliphatic halogen (alkylating, direct acting agents)	Mutations, oxidative stress
2. Epoxides and aziridines (alkylating, direct acting agents)	Cell proliferation, cell death
3. Hydrazine (alkylating, direct acting agents)	Oxidative stress
4. Unspecific mutagens	Oxidative stress, mutations
5. Nitro aromatics (DNA adducts, indirect acting agents)	Mutations, chromosomal changes
6. Non-mutagens	Cell proliferation, oxidative stress
7. Primary aromatic amines (DNA adducts, indirect acting agents)	Strand breaks, mutations
8. Quinones (alkylating, direct acting agents)	Mutations, chromosomal changes

*Carcinogens with the same structural alerts were grouped. Each group was linked with their corresponding literature profile. The most and second most common MOA category for each group is shown*.

**FIGURE 4:**
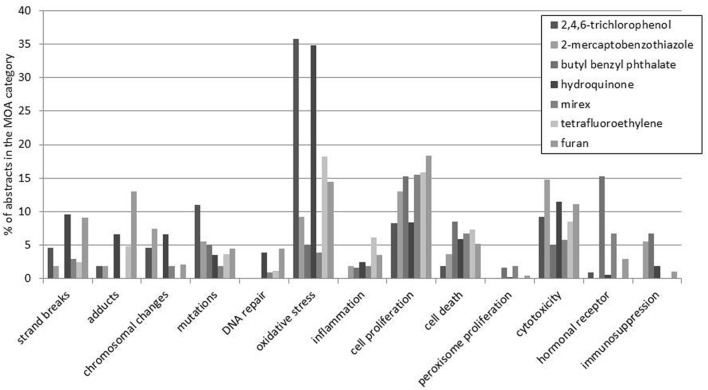
**Seven non-mutagenic rat carcinogens of the hematopoietic system were investigated in more detail**. The figure shows 13 selected MOA categories and the literature distribution over these classes for carcinogens affecting the hematopoietic system in rats.

The literature patterns generated by the CRAB-tool can provide new information of potential interest that can be used to form new hypotheses. When the output from the QSAR analysis was linked with information on the target organs affected, we found that the group of carcinogens affecting the hematopoietic system included a larger proportion of non-mutagens (7 of 19 carcinogens with QSAR data) compared to the other organs. The literature patterns of these seven non-mutagens were analyzed in more detail (**Figure 4**) and we found that the most common endpoints studied for these carcinogens were oxidative stress, cell proliferation, and cytotoxicity, which are all non-genotoxic effects. Interestingly, the literature concerning five of these non-mutagens (2,4,6-Trichlorophenol, Butyl benzyl phthalate, Hydroquinone, Mirex, and Furan) had data classified in the category of hormonal receptor-mediated effects. This result is also in line with what is known about some of these compounds (Ma et al., 2011; Upson et al., 2013; Alam and Kurohmaru, 2016).

## Discussion

In this study, we tested the idea of combining the QSAR method with a text-mining approach to generate more detailed information regarding the relationship between chemical structures and carcinogenic mechanisms (MOAs). The literature of 96 rat carcinogens was analyzed using the text mining-based CRAB tool (Korhonen et al., 2009, 2012; Guo et al., 2014). QSAR models were used to predict mutagenicity and structural alerts for 75 of these carcinogens. The chemicals were grouped based on target organ, mutagenicity and structural alerts, and literature profiles were generated for each chemical group with the aim to discover new patterns in data that connect target organs, chemical structures, and carcinogenic MOAs.

The text-mining analysis showed that the mutation endpoint was frequently studied in connection with most of the 96 rat carcinogens, particularly in relation to lung and skin carcinogens. This is not surprising as mutagenicity is known to have a central role in carcinogenesis. In addition, the mutation endpoint is widely used in studies of carcinogens and in screening tests of mutagenicity (Mortelmans and Zeiger, 2000). By using QSAR models we also found that the groups of carcinogens affecting the liver and the hematopoietic system in rats included a large proportion of non-mutagens. These data are in line with a previous study of 522 carcinogens (Ashby and Paton, 1993), where it was shown that these organs were partly affected by carcinogens without reactive molecular sites. The same study also showed that rat lung and skin carcinogens included mainly reactive chemicals (Ashby and Paton, 1993).

Data patterns related to rare carcinogenic endpoints may also be of interest, e.g., regarding non-genotoxic chemicals for which detailed carcinogenic mechanisms may not be known. By using the text-mining approach to compare groups of chemicals new data patterns of potential importance can be found. In the current study, we found that immunosuppression was frequently mentioned in the literature concerning rat carcinogens affecting the hematopoietic system. This is an interesting finding, which is also in line with the known mechanisms of human carcinogens affecting this organ (Adamson and Seiber, 1981; IARC, 2015). An association between immunosuppressant drugs and development of cancer in the hematopoietic system (lymphomas) in humans has also been shown previously (Bugelski et al., 2010). However, as the value of the rodent carcinogenicity assay in predicting human toxicity caused by immunosuppressants has been questioned (Bugelski et al., 2010) it would be of interest to apply the same method on a set of human carcinogens affecting the hematopoietic system.

Another finding concerning carcinogens of the hematopoietic system was a relatively large proportion of literature linked to hormonal effects, compared with the other organs. Although the findings were based on only a few rat carcinogens this result may indicate a potentially important mechanism for cancer development in this organ, possibly also for humans. Although there are articles reporting on potential links between hormonally active substances and cancer of the hematopoietic system in humans (Traversa et al., 1998; Poynter et al., 2013; Leal et al., 2016), the aetiologies of this cancer type are still unclear (Laurier et al., 2014). More research is required to support these findings and it would, e.g., be of interest to evaluate the structures of the chemicals in more details and investigate potential links with hormonal receptors. In addition, human carcinogens targeting this organ should be analyzed using the same approach.

When the rat carcinogens were organized into groups based on their proposed structural alerts, we found that the literature of carcinogens with predicted mutagenic structural alerts reported more frequently about genotoxic effects compared to non-mutagenic carcinogens. This comparison confirms that the outcomes of the two methods are consistent. Our initial idea was that more detailed information regarding structural alerts linked to text mining-generated information could provide new data patterns of potential interest. This approach could be particularly useful to increase the knowledge about how non-genotoxic compounds act, e.g., in a certain organ. More detailed structural information could be important because the knowledge about how the chemical structures of these compounds link to biological effects, on a mechanistic level, is still weak. Furthermore, a problem in current non-animal based cancer testing is the lack of reliable systems to detect non-genotoxic carcinogens (Benigni et al., 2013). Thus, development of new approaches to study, e.g., non-mutagenic carcinogens is important to improve future testing strategies.

Although QSAR models have proven useful in predicting mutagens, the method is more challenging for non-genotoxic carcinogens (Silva Lima and Van der Laan, 2000; Benigni et al., 2013; Luijten et al., 2016). There are several explanations for this difference, e.g., a better mechanistic understanding of how mutagenic compounds cause cancer, compared to non-genotoxic carcinogens. Furthermore, the databases used for QSAR contain more data on mutagenic carcinogens, which makes the basis for analysis stronger, leading to more robust predictions for mutagens (Benigni et al., 2013). Another more general difficulty related to non-genotoxic carcinogens is that these compounds may target specific organs, often depending on organ-specific metabolic mechanisms (Silva Lima and Van der Laan, 2000). As these characteristics can be species-specific, the human relevance of certain non-mutagenic mechanisms may be unclear. Predicting metabolic induction of enzymes such as cytochromes P450 using computational approaches (Kirchmair et al., 2015) could be useful to identify chemicals with potential to cause tumors in, e.g., the rodent liver (Graham and Lake, 2008).

In this study we have combined QSAR data with text mining-generated literature profiles of carcinogenic MOAs to generate new patterns in data to explain the link between chemical structure and carcinogenic effects. This approach could be valuable in studies of non-mutagens, where more knowledge about structure and activity relationships is needed. The overall strategy, using these two methods in combination, also needs further evaluation, e.g., by including additional non-mutagens in the analysis and to further test its usefulness, maybe also as a predictive approach.

## Author Contributions

IS conceived the original idea, designed and performed research, analyzed results, wrote the paper. GP designed and performed research, analyzed results and wrote the paper. Both authors approved the submitted manuscript.

## Conflict of Interest Statement

The authors declare that the research was conducted in the absence of any commercial or financial relationships that could be construed as a potential conflict of interest.

## Footnotes

1 http://www.ncbi.nlm.nih.gov/pubmed

2 http://ntp.niehs.nih.gov/index.cfm

3 http://www.ncbi.nlm.nih.gov/pubmed

4 http://www.vega-qsar.eu/
